# Liquid-like droplet formation by tumor suppressor p53 induced by multivalent electrostatic interactions between two disordered domains

**DOI:** 10.1038/s41598-020-57521-w

**Published:** 2020-01-17

**Authors:** Kiyoto Kamagata, Saori Kanbayashi, Masaya Honda, Yuji Itoh, Hiroto Takahashi, Tomoshi Kameda, Fumi Nagatsugi, Satoshi Takahashi

**Affiliations:** 1 Institute of Multidisciplinary Research for Advanced Materials, Tohoku University, Katahira 2-1-1, Aoba-ku, Sendai 980-8577 Japan; 2 Department of Chemistry, Graduate School of Science, Tohoku University, Sendai, 980-8578 Japan; 3 Artificial Intelligence Research Center, National Institute of Advanced Industrial Science and Technology (AIST), Koto, Tokyo 135-0064 Japan

**Keywords:** DNA-binding proteins, Imaging, Microscopy, Optical spectroscopy, DNA, Proteins, Biophysics, Biochemistry, Protein aggregation

## Abstract

Early *in vivo* studies demonstrated the involvement of a tumor-suppressing transcription factor, p53, into cellular droplets such as Cajal and promyelocytic leukemia protein bodies, suggesting that the liquid-liquid phase separation (LLPS) might be involved in the cellular functions of p53. To examine this possibility, we conducted extensive investigations on the droplet formation of p53 *in vitro*. First, p53 itself was found to form liquid-like droplets at neutral and slightly acidic pH and at low salt concentrations. Truncated p53 mutants modulated droplet formation, suggesting the importance of multivalent electrostatic interactions among the N-terminal and C-terminal domains. Second, FRET efficiency measurements for the dimer mutants of p53 revealed that distances between the core domains and between the C-terminal domains were modulated in an opposite manner within the droplets. Third, the molecular crowding agents were found to promote droplet formation, whereas ssDNA, dsDNA, and ATP, to suppress it. Finally, the p53 mutant mimicking posttranslational phosphorylation did not form the droplets. We conclude that p53 itself has a potential to form droplets that can be controlled by cellular molecules and by posttranslational modifications, suggesting that LLPS might be involved in p53 function.

## Introduction

Tumor suppressor p53 is a multifunctional transcription factor that induces cell cycle arrest, DNA repair or apoptosis upon binding to its target DNA sequence. In 50% of human cancers, mutations on p53 are found to hamper its binding to the target sequence. Accordingly, extensive investigations have been conducted to characterize the functions as well as malfunctions of p53. However, an important aspect of p53, namely its involvement in liquid-like droplets, is still largely unresolved. In fact, p53 has long been known to be uptaken into cellular droplets such as Cajal and promyelocytic leukemia protein (PML) bodies. In this report, we describe that p53 itself can form liquid-like droplets upon the control of solution conditions, suggesting a possible involvement of the p53 droplets in the cellular environment.

The primary function of p53 is the accommodation of various posttranslational modifications, termed activation, which in turn triggers the search for and the binding to its target DNA sequence, leading to the expression of downstream genes^1^. p53 is composed of the N-terminal (NT) (residues 1–95), the core (95–293), the linker (293–326), the tetramerization (Tet) (326–357), and the C-terminal (CT) (357–393) domains. p53 slides along nonspecific DNA by attaching the CT domain to the DNA and by hopping the core domain^2–4^. The sliding of p53 occurs in two modes^5,6^, in which the CT, core and linker domains are differently in contact with the DNA^7^. The recognition efficiency of the target sequence by the sliding p53 is low, but is enhanced by the activation of p53^8^. Furthermore, the sliding p53 can transfer from one DNA strand to another using the CT domain^9^. In addition, Molecular Dynamics (MD) simulations provided detailed insights into the molecular events involving p53^10–12^. Overall, significant information has been accumulated on this protein’s target search mechanisms.

p53 is also known to form amyloid-like aggregates, which decrease its anticancer functions. The core and Tet domains of the wild-type p53 have a high tendency to aggregate in a temperature-sensitive manner^13–18^. Some mutations of the core domain are relevant to cancerization by destabilizing the domain’s tertiary structure and by promoting the aggregation of p53^19–24^. The removal of Zn^2+^ from the core domain enhanced aggregation due to destabilization of the domain^23,25^. Furthermore, p53 was reported to co-aggregate with its homologs^26^. Accordingly, accumulated evidence has demonstrated that the destabilization of the p53 folded domains leads to amyloid formation, harming the protein’s functionality^14,15,21,23^.

Apart from the solid aggregates of p53, increasing evidence suggests that liquid-like droplet formation, sometimes referred to as liquid-liquid phase separation (LLPS), may positively participate in p53 regulation. Various types of liquid-like droplets composed of proteins with disordered sequences and RNAs have been reported to participate in stress response, transcription, and signal transduction^27,28^. p53 is uptaken into nuclear bodies such as PML and Cajal bodies in conditions of cell stress^29–31^. The localization of p53 in the PML body enhances transcriptional regulations of p53^30,32,33^ and regulates the expression of nuclear factors in activated T-cells^34^. p53 in the PML body is protected from the degradation mediated by MDM2 ubiquitin ligase and proteasome^35,36^. Inhibition of the co-localization of p53 and survival of motor neuron protein in the Cajal body may cause spinal muscular atrophy^37^. The posttranslational modification of p53 is enhanced in the PML body^38–41^. Accordingly, the *in vivo* studies strongly suggested that LLPS is inherently involved in p53 function in cells.

In this study, we investigated whether p53 itself forms the liquid-like droplets in the *in vitro* conditions. While p53 does not possess the low complexity regions frequently found in proteins demonstrating LLPS, the presence of multiple domains might facilitate droplet formation by allowing multivalent interaction among p53^42–46^. We found that the purified p53 itself forms the liquid-like droplets and we dissected the domains responsible for the droplet formation using a series of biophysical approaches. In particular, we found that droplet formation is dependent on phosphorylation, suggesting the involvement of the droplets in the activation mechanism of p53.

## Results

### p53 itself forms spherical droplets at neutral and slightly acidic pH and at low salt concentrations

As a preliminary experiment, we investigated whether p53 itself forms the liquid-like droplets. We used a thermostable mutant termed a full-length pseudo wild-type p53 (FL-p53) to reduce the irreversible aggregation found in wild-type p53^5^. At a FL-p53 concentration of 100 μM and pH 7.9^5–9^, we detected an increase in light scattering at 350 nm. However, imaging of the solutions using a differential interference contrast (DIC) microscope could not identify any μm-sized assemblies. The results suggested the formation of scattering objects with sizes of less than a micrometer, which is consistent with the observation for wild-type p53 reported by Safari *et al*.^47^. The scattering might be caused either by liquid-like droplets or by small solid particles. In order to further examine the scattering objects, we searched for different solution conditions that could form larger droplets. Since p53 contains many charged residues in its disordered domains, we varied pH and salt concentration to tune the total net charge and the electrostatic interactions, respectively.

**Figure 1:**
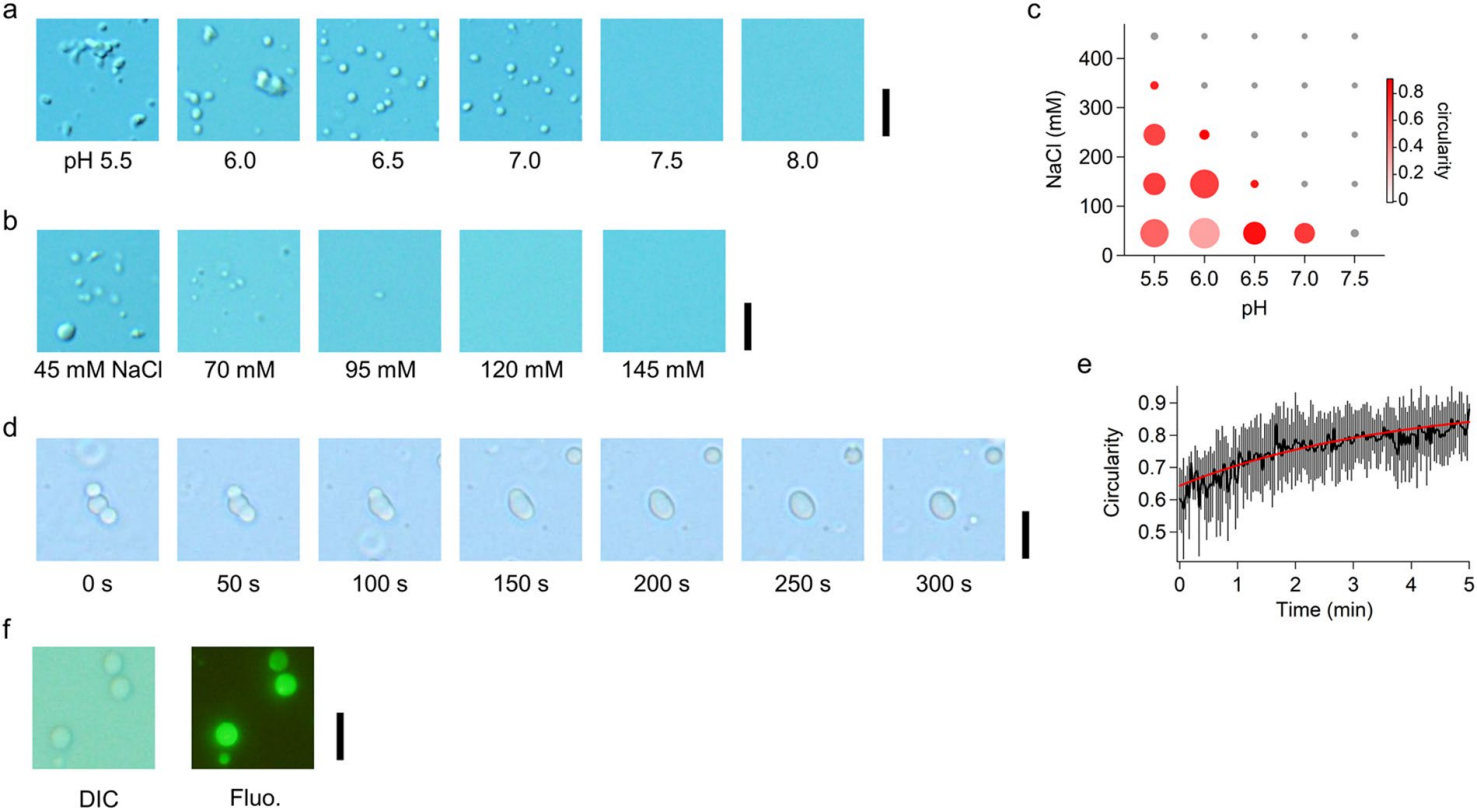
FL-p53 droplet formation is sensitive to pH and salt concentration. (**a**) DIC images of the FL-p53 solution in the presence of 45 mM NaCl at various pHs. (**b**) DIC images of the FL-p53 solution at pH 7.0 in the presence of various concentrations of NaCl. (**c**) 2D plot showing the pH and salt concentration dependences of FL-p53 droplet formation. The size of the circles is proportional to the OD_350_. The color of the circles represents the average circularity of the droplets. (**d**) Time course of a typical fusion event of three FL-p53 droplets into a single droplet observed at pH 7.0 in the presence of 45 mM NaCl and 150 mg/mL dextran. FL-p53 concentration was 12 μM in the experimental results shown in panels (**a**–**d**). (**e**) Time course of the average circularity of the fusion events. The bars denote the standard deviation of 6 fusion events. The red curve is the best-fitted curve by a single exponential. (**f**) DIC and fluorescence images of the p53 droplets formed by 0.12 μM Alexa488-labeled p53 and 12 μM non-labeled FL-p53 at pH 7.0 in the presence of 45 mM NaCl and 150 mg/mL dextran. Scale bars in panels (a,b,d,f) represent 10 μm.

We first examined the effect of pH in droplet formation for 12 μM FL-p53 in the presence of 45 mM NaCl (Fig. 1a). We measured the p53 solutions after 10-fold dilution of a stock solution containing 450 mM NaCl at pH 7.5 into different buffers. Spherical droplets with apparent cross sections of 0.2–4 μm^2^ were observed at pH 7.0 (Supplementary Fig. S1). At lower pHs, the sizes of the clusters became larger. Non-spherical and larger clusters composed of smaller spherical droplets became dominant at pH 5.5. In contrast, neither the droplets nor the clusters were observed at pH 8.0. Since the estimated pI of FL-p53 was 6.4, the results suggest that the decrease in electrostatic repulsion at the slightly acidic conditions might promote droplet formation. We next examined the effect of salt concentration at pH 7.0 in the presence of 12 μM FL-p53 and observed the disappearance of the spherical droplets at higher salt concentrations (Fig. 1b). Accordingly, the droplet formation of p53 was sensitive to both pH and salt concentration, suggesting the importance of electrostatic interactions.

In order to further examine the electrostatic effect on droplet formation, we characterized droplets and clusters of FL-p53 by analyzing their shapes in the DIC images and by measuring their scattering intensities at 350 nm at various pHs and salt concentrations (Fig. 1c). In the top-right triangle of the two-dimensional (2D) plot showing the pH and [NaCl] dependencies of the droplet formation, the scattering intensity measured as the absorbance at 350 nm (OD_350_) equaled to 0, indicating the absence of droplets. In contrast, OD_350_ grew in the bottom-left triangle of the 2D plot (Size of the circles in Fig. 1c). Among them, for all the data obtained at pH 5.5 and at pH 6.0 in the presence of 45 mM NaCl, the circularity of the droplets and/or the clusters (defined as 4π*S*/*L* where *S* and *L* are the cross section and the perimeter, respectively) was broadly distributed between 0.2 and 1.2, showing that the non-spherical clusters were dominant over the spherical droplets (Color of the circles in Fig. 1c and Supplementary Fig. S2). Interestingly, in the boundary between the top right and bottom left triangles in the 2D plot, the main circularity peak appeared around 1, showing that spherical droplets were dominant (* in Supplementary Fig. S2). These results demonstrated that the formation of circular p53 droplets occurs only in narrow solution conditions. If the attractive electrostatic interaction is too weak, droplets are not formed. If the electrostatic interaction is too strong, apparent aggregation of the spherical assemblies should be observed. Thus, an appropriate intermolecular interaction among p53 molecules is necessary for the circular droplet formation.

In order to further confirm the liquid-like p53 droplet formation, we investigated the droplets’ fluidity and the density of FL-p53 within them. To confirm the fluidity of the droplets, we observed the fusion process of multiple droplets. In the trial experiments conducted at pH 7.0 and at 45 mM NaCl, we faced two obstacles that hampered observation: the adsorption of droplets to the coverslip and the rare collision of two droplets. In order to prevent the adsorption, we coated the coverslip using 2-methacryloyloxyethyl phosphorylcholine (MPC) polymer^48^. We also added 150 mg/mL dextran to increase the number of droplets and their collision events. As we will discuss later, the addition of dextran promotes droplet formation by the molecular crowding effect. In the example shown in Fig. 1d, three droplets collided simultaneously and fused into a single circular droplet. The average time constant for the fusion process, obtained by fitting the time course of the average circularity with a single exponential, was 3.7 ± 1.0 min (*N* = 6) (Fig. 1e). Finally, we examined FL-p53 density in the droplet by measuring the fluorescence intensity of droplets formed in the presence of 1% of labeled p53, whose 292 C was modified with a fluorescent dye, Alexa488. The fluorescence intensity inside the droplet was significantly higher than that outside the droplet corresponding to the solution (Fig. 1f). In addition, fluorescence intensity in single droplets was uniform throughout the diameter, suggesting a homogeneous distribution of p53 in the droplet. Accordingly, we conclude that the circular droplets observed at neutral and slightly acidic pHs are highly fluidic and are composed of high concentrations of p53.

### p53 droplet formation is mediated by the NT and CT disordered domains

**Figure 2:**
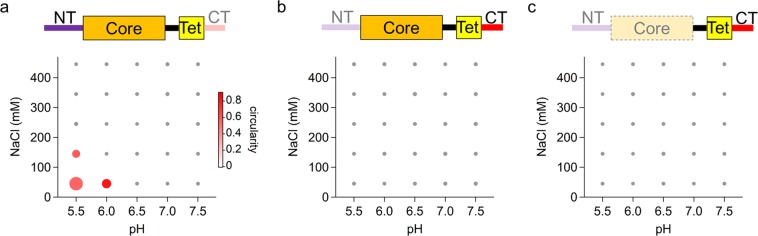
Deletion of the disordered domains hindered p53 droplet formation. The 2D plots representing droplet formation of the NTCoreTet (**a**), CoreTetCT (**b**), and TetCT mutants (**c**) at different pHs and salt concentrations. The size and color of the circles represent OD_350_ and the average circularity of the droplets, respectively. The concentration of each mutant was 12 μM. In the primary structure of mutants presented at the top of each plot, rectangles and lines denote folded and disordered domains, respectively. The shaded regions in the primary structures are the deleted domains.

In order to identify the domains that participate in droplet formation, we investigated three deletion p53 mutants: the NTCoreTet mutant (residues 1–363) lacking the CT domain, the CoreTetCT mutant (residues 94–393) lacking the NT domain, and the TetCT mutant (residues 293–393) lacking the NT and core domains^6^ (Fig. 2). The three mutants were purified as described in our previous report^6^. We investigated droplet formation of the deletion mutants at 12 μM at varying pH and salt concentrations and in the absence of dextran. Considering the NTCoreTet mutant, the region of droplet formation in the 2D plot was narrowed and significantly shifted to the lower-left corner when compared to that of FL-p53 (Figs. 1c and 2a). This result suggests that droplet formation is mediated by the CT domain. Furthermore, no droplets were observed for the CoreTetCT and TetCT mutants in any of the examined pH and salt concentrations, suggesting the participation of the NT domain in droplet formation (Fig. 2b,c).

Considering that the NT and CT domains respectively possess highly negative (−13.7) and positive (+4.7) net charges (Supplementary Table S1 and text), the electrostatic interaction between these domains might be the key interaction that mediates p53 droplet formation. In fact, p53 droplet formation was significantly suppressed at higher concentrations of NaCl (Fig. 1c and Supplementary Fig. S3a). In addition, the net charge of the entire p53 molecule might also be important for droplet formation, since the NTCoreTet mutant formed droplets at pHs around its pI of 5.4, similarly to the observed FL-p53 droplet formation around its pI of 6.4. We also examined the effect of 1,6-hexanediol on p53 droplet formation, since it is known to weaken the non-electrostatic interactions such as cation-π and π-π interactions (Supplementary Fig. S3b). The effect was less significant than that observed for other proteins possessing the low complexity sequence region^49,50^, implying a smaller importance of non-electrostatic interactions in p53 droplet formation. Overall, the current data demonstrated that the electrostatic interaction between the NT and CT disordered domains mediates p53 droplet formation.

### p53 conformation in solution and in droplets

**Figure 3:**
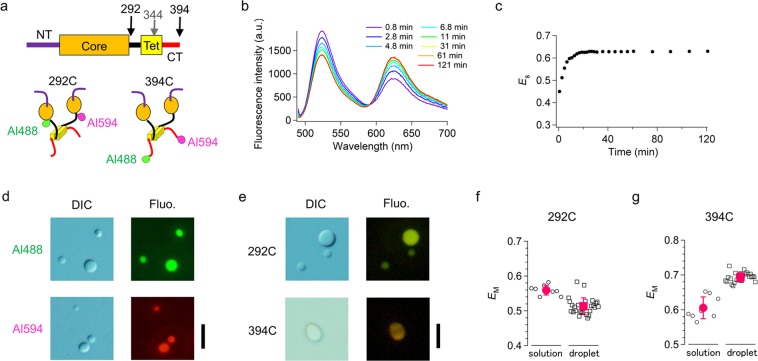
Structural characterization of the labeled dimer p53 mutants in solution and in FL-p53 droplets using FRET. (**a**) Schematic diagram of the primary and tertiary structures of the dimer mutant L344A. The cysteines were introduced either at position 292 or at position 394 for labeling with Alexa488 and Alexa594. (**b**) Fluorescence spectral changes observed after mixing the Alexa488-Alexa488 dimer and the Alexa594-Alexa594 dimer, both labeled at 292 C at pH 7.9 in the presence of 50 mM KCl. The different times describe the periods after mixing. (**c**) Time course of the spectrum-based FRET efficiency, *E*_S_, after mixing the Alexa488-Alexa488 dimer and the Alexa594-Alexa594 dimer labeled at 292 C. The data shown in panel (b) were used for *E*_S_ calculation. (**d**) DIC and fluorescence microscopic images of the samples containing 0.12 μM of the Alexa488-Alexa488 dimer labeled at 292 C and 12 μM of FL-p53 (upper panels) at pH 7.0 in the presence of 45 mM NaCl and 150 mg/mL dextran. The corresponding DIC and fluorescence images prepared by adding the Alexa594-Alexa594 dimer labeled at 292 C in the same condition are shown in the lower panels. The fluorescence images were obtained through excitation at 470–490 nm for Alexa488 and at 520–550 nm for Alexa594. (**e**) DIC and fluorescence microscopic images of the sample after adding the Alexa488-Alexa594 dimer labeled at 292 C to the preformed FL-p53 droplets (upper panels) at pH 7.0 in the presence of 45 mM NaCl and 150 mg/mL dextran. The corresponding DIC and fluorescence images prepared by adding the Alexa488-Alexa594 dimer labeled at 394 C in the same conditions are shown in the lower panels. The fluorescence image was obtained by excitation at 470–490 nm. (**f**) Comparison between the apparent FRET efficiencies based on the microscopic data, *E*_M_, of the Alexa488-Alexa594 dimer labeled at 292 C in solution (left) and in the droplets (right). (**g**) Comparison between the *E*_M_ for the Alexa488-Alexa594 dimer labeled at 394 C in solution (left) and in the droplets (right). Error bars are the standard deviation. Scale bars in panels (d,e) represent 10 μm.

With the objective of examining the structural properties of p53 in solution and in droplets, we measured the fluorescence resonance energy transfer (FRET) between two fluorophores labeled at two residues of p53. We prepared two dimer p53 mutants based on L344A, causing the dimerization of p53, so as to prevent the heterogeneous labeling of two fluorophores to four labeling sites in one tetramer of FL-p53. One of the two L344A mutants maintains the K292C substitution located at the end of the core domain in FL-p53. In contrast, the other mutant eliminates the cysteine at position 292 (C292K) but possesses a 394 C extension at the end of the CT domain (Fig. 3a). Firstly, we labeled the dimer mutants either with Alexa488 or with Alexa594 at a labeling ratio of approximately one per monomer (Supplementary Table S2). Secondly, we mixed 10 nM of the 488–488 dimer with 80 nM of the 594–594 dimer and initiated monomer exchange in order to generate the 488–594 dimer. Since the mixing ratio between the two samples was 8, the mixed sample after complete exchange should contain 19.8% of the 488–594 dimer, 1.2% of the 488–488 dimer, and 79.0% of the 594–594 dimer. Accordingly, we could minimize the amount of 488–488 dimer that would affect the measured FRET efficiency between Alexa488 and Alexa594 in the 488–594 dimer. We measured the exchange reaction of the dimer mutants labeled at 292 C after manual mixing of the two samples in a solution containing 50 mM KCl at pH 7.9 by using a fluorescence spectrometer, and detected the reduction of donor fluorescence and the increase of acceptor fluorescence simultaneously (Fig. 3b). FRET efficiency, measured in accordance to the spectroscopic data (*E*_s_), increased throughout the exchange reaction progress and was saturated after 20 min (Fig. 3c; see Eq. 1). We observed a similar exchange reaction for the dimer mutant labeled at 394 C (Supplementary Fig. S4). The FRET efficiency for this dimer, estimated from the mean theoretical distance between two dyes assuming a Gaussian chain for disordered regions, was 0.76 for the 292^nd^ residues and 0.72 for the 394^th^ residues, and was consistent with the *E*_s_ values of 0.63 and 0.58, respectively (Supplementary text). Accordingly, the FRET efficiency reflected the structural information of p53.

We next examined the uptake of dimer mutants into the p53 tetramer droplet formed by FL-p53 using DIC and fluorescence microscopies. We incubated FL-p53 at 12 μM and pH 7.0 and added the 488–488 dimer or the 594–594 dimer at 0.12 μM (Fig. 3d). The fluorescence intensity of the 488–488 or 594–594 dimers in the droplets was significantly larger than that of the solution, indicating the uptake of the p53 dimer into the FL-p53 droplets. The uptake of the dimer into the droplets took several minutes. The fluorescence intensity distribution within each of the droplets was uniform, suggesting a homogeneous distribution of dimers in the droplets.

In order to verify structural changes, we measured FRET efficiency for the 488–594 dimer labeled at 292 C in the droplets. We first mixed the 488–488 and the 594–594 dimers in solution and incubated for 20 min to generate the 488–594 dimer. Since the mixing ratio between the 594–594 dimer and the 488–488 dimer was 5, we assumed the presence of 27.8% of the 488–594 dimer, 2.8% of the 488–488 dimer and the 69.4% of the 594–594 dimer. We then added the mixed solution to the preformed FL-p53 droplets. We used a color-sensitive camera to separate donor and acceptor fluorescence intensities at a wavelength cutoff of 580 nm. Fluorescence images obtained for the dimer mutant labeled at 292 C clearly demonstrated that the 488–594 dimer was uptaken by the FL-p53 tetramer droplet (Fig. 3e). The apparent FRET efficiency determined from the microscope image, *E*_M_, was obtained for each droplet (see Eq. 2). The average droplet *E*_M_ was 0.51 ± 0.02 (*N* = 25, Fig. 3f). The average *E*_M_ for the same sample obtained by measuring donor and acceptor fluorescence intensities in the absence of FL-p53 was 0.56 ± 0.01 (Fig. 3f). Thus, the distance between the labeled 292^nd^ residues in the dimer, corresponding to the distance between the core domains, was slightly larger in the droplet when compared to that in the solution. In contrast, the average determined *E*_M_ from the corresponding experiments that used dimer mutants labeled at 394 C shifted from 0.61 ± 0.03 in the solution to 0.69 ± 0.01 in the droplets (*N* = 24), suggesting that the distance between the ends of the CT domains was slightly smaller in the droplets (Fig. 3g). Accordingly, the p53 in droplets adopted a new tertiary structure forming interactions with the adjacent molecules.

### Target binding of p53 dissolved from the droplets

**Figure 4:**
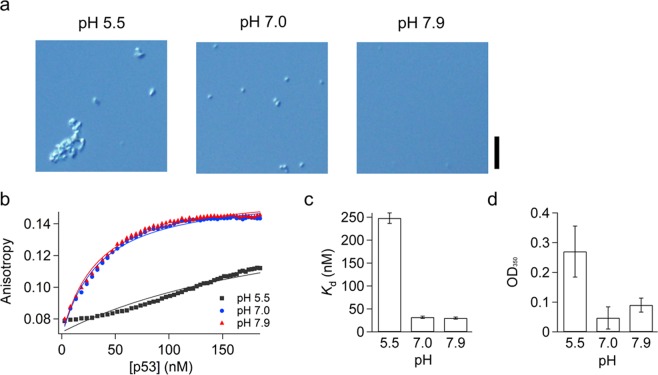
Target binding of FL-p53 dissolved from the droplets. (**a**) DIC images of 12 μM FL-p53 in the presence of 45 mM NaCl and 2 mM MgCl_2_ at pH 5.5, 7.0, and 7.9. A scale bar represents 10 μm. (**b**) Fluorescence anisotropy changes for the association of FL-p53 to the specific double-stranded DNA sequence conjugated with 6-FAM. The titration of FL-p53 was conducted in the presence of 90 mM NaCl and 2 mM MgCl_2_ at pH 7.9 after 5-fold dilution of the FL-p53 solutions incubated in the three pH conditions shown in panel (a). Solid curves represent the best-fitted curves based on the equation assuming the one-to-one binding^5^. (**c**) *K*_d_ values of p53 for the specific double-stranded DNA sequence. The results obtained for p53 incubated at pH 5.5, 7.0 and 7.9 were compared. (**d**) Scattering intensity at 350 nm of the p53 solutions in the presence of 90 mM NaCl and 2 mM MgCl_2_ at pH 7.9 after 5-fold dilution of the FL-p53 solutions incubated in the different pHs shown in panel (a). Error bars in panels (c,d) are the fitting errors and the standard errors of three independent measurements, respectively. p53 concentrations denoted in panels (b,c) were per tetramer.

We next investigated whether the target binding of p53 is affected by the droplet formation or not. If the droplet formation of p53 involves irreversible denaturation, the target search and binding of p53 once experienced the droplet formation should be inhibited. In contrast, if the droplet formation of p53 is reversible, p53 dispersed in solution from the droplets should be able to search for and bind to the target DNA. We here examined the binding of p53 to 30 bp double-stranded DNA containing the target sequence, dsDNA_sp_, in the condition where p53 is dispersed at pH 7.9 and 90 mM NaCl after incubating the samples at three pH conditions. The DIC images in Fig. 4a showed that FL-p53 in the presence of 45 mM NaCl is dispersed at pH 7.9 but forms spherical droplets and non-spherical clusters at pH 7.0 and 5.5, respectively. The target binding of the three samples was then monitored as the change of the fluorescence anisotropy of FAM conjugated with dsDNA_sp_^5^. The anisotropy curve of p53 once formed the spherical droplets at pH 7.0 was almost identical to that of p53 dispersed in the solution at pH 7.9 (Fig. 4b). The dissociation constants (*K*_d_) of p53 with and without experiencing the droplet formation obtained by fitting the anisotropy curves with the equation based on the one-to-one binding^5^ were identical (Fig. 4c). The results demonstrated that p53 can maintain the affinity to dsDNA_sp_ after experiencing the droplets and that the droplet formation at pH 7.0 is reversible.

In contrast to the spherical droplets formed at pH 7.0, p53 once experienced the non-spherical clusters could not restore the binding to dsDNA_sp_. The anisotropy curve for the sample incubated at pH 5.5 did not follow the typical one-to-one binding behavior and demonstrated the significant reduction of the affinity to dsDNA_sp_ (Fig. 4b). The dissociation constant roughly estimated by fitting the titration curve using the one-to-one binding model was 8-fold larger than those incubated at pH 7.0 and 7.9 (Fig. 4c). The scattering intensity at 350 nm obtained in the solution at pH 7.9 and 90 mM NaCl was larger for the sample once incubated at pH 5.5 than those incubated at pH 7.0 and 7.9, suggesting that the non-spherical clusters could not be dissolved completely (Fig. 4d). The results demonstrated that the non-spherical clusters formed at pH 5.5 cannot be reversibly dissolved into the dispersed and functional tetramers and that the clusters are distinct from the liquid droplets formed at pH 7.0.

### Droplet formation of p53 is regulated by crowding, nucleic acids, and posttranslational modification

**Figure 5:**
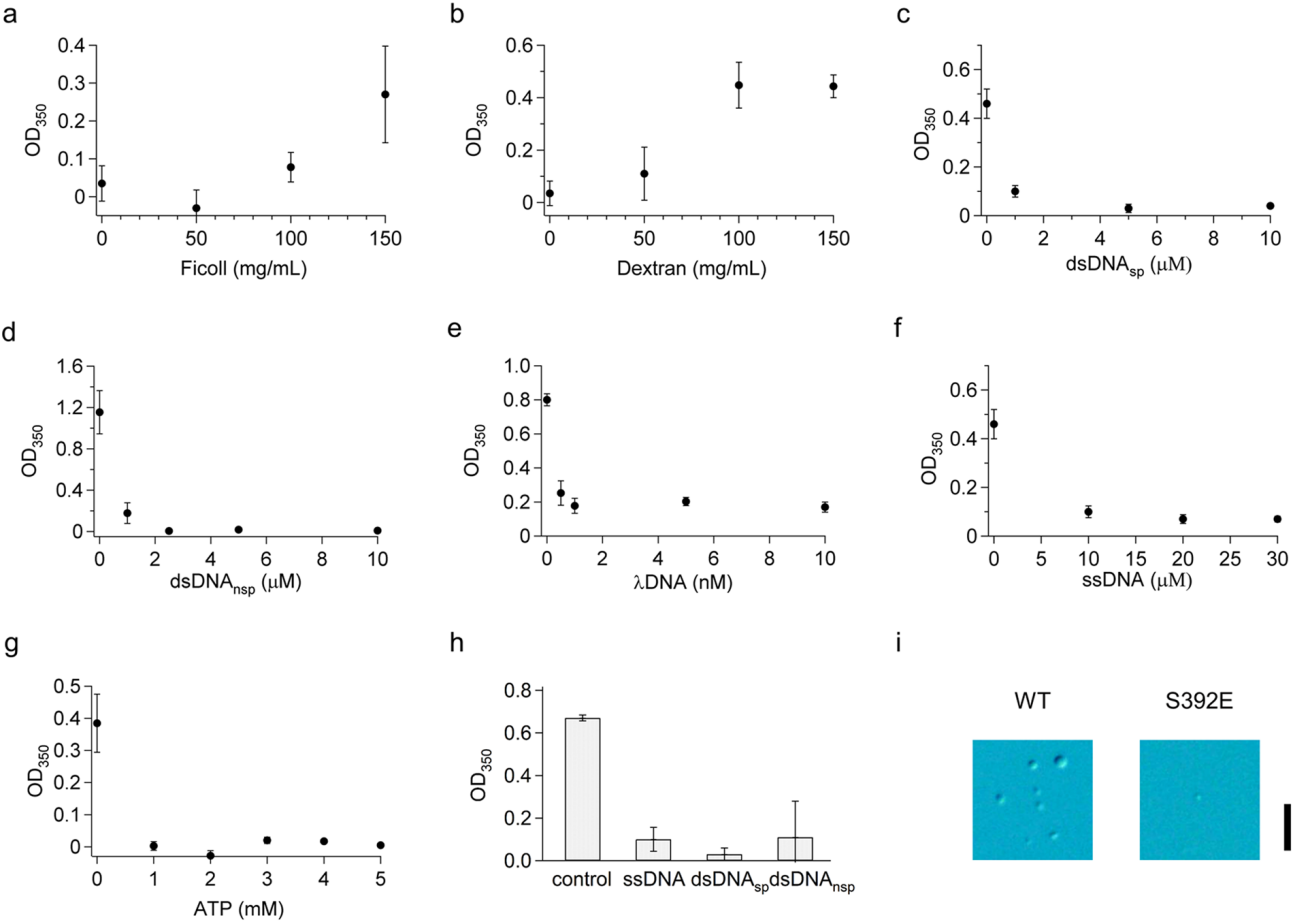
Molecular crowding promoted, while nucleic acids and a posttranslational modification suppressed p53 droplet formation. (**a**) Ficoll- and (**b**) Dextran-concentration dependence of p53 droplet formation detected as the scattering intensity at 350 nm in a solution containing 145 mM NaCl at pH 7.0. (**c**–**g**) DNA- and ATP-concentration dependence of p53 droplet formation detected as the scattering intensity at 350 nm in a solution containing 45 mM NaCl at pH 7.0. Panels (c–g) represent data from the addition of the specific double-stranded DNA sequence, of the non-specific double-stranded DNA, of λDNA, of single-stranded DNA, and of ATP, respectively. (**h**) Comparison of the effects of different DNAs in p53 droplet formation detected at pH 7.0 in the presence of 145 mM NaCl and 150 mg/mL dextran. The DNA concentration was 100 μM. Control denotes the result obtained in the absence of DNA. (**i**) Effect of the S392E mutation (mimicking posttranslational phosphorylation) on p53 droplet formation at pH 7.0 and in the presence of 45 mM NaCl. DIC images of FL-WT and S392E are displayed. Scale bar represents 10 μm. In panels (a–h), the error bars represent SEM of at least three measurements.

We investigated the molecular crowding effect on FL-p53 droplet formation by adding ficoll or dextran as crowders. In the absence of the crowders, scattering was not detected for a solution at pH 7.0 in the presence of 145 mM NaCl and 10 μM FL-p53 (Fig. 5a). When using both ficoll and dextran, scattering was detected at crowder concentrations higher than 100 mg/mL (Fig. 5a,b). The dependence on salt concentration when using of 150 mg/mL ficoll confirmed that the electrostatic interaction was still important for the formation of the p53 droplet, as observed in the absence of ficoll (Supplementary Fig. S3a). The results demonstrated that the size exclusion effect promoted the p53 droplet formation.

We next examined the effect of DNA and ATP on p53 droplet formation in a solution containing 10 μM FL-p53 and 45 mM NaCl at pH 7.0. The addition of the target sequence, dsDNA_sp_, suppressed droplet formation even at 1 μM, which is a substoichiometric concentration for p53 (Fig. 5c). The results suggested that the specific p53/dsDNA_sp_ complex, in which p53 is bound to DNA via the core domain^51–55^, did not form droplets probably due to the electrostatic repulsion between DNAs present in the complexes. Similarly, the non-specific 30-bp double-stranded DNA, dsDNA_nsp_, suppressed droplet formation at 2.5 μM, suggesting that the non-specific p53/dsDNA_nsp_ complex, in which p53 is bound to DNA via the CT and core domains^3,5,6^, did not form the droplet (Fig. 5d). λDNA possessing a 48.5-kbp non-specific sequence suppressed droplet formation more efficiently (Fig. 5e). Furthermore, 30-bp single-stranded DNA, ssDNA, and ATP suppressed droplet formation (Fig. 5f,g), suggesting that the positive charges of the CT domain might be screened by the negative charges of ssDNA or ATP. Even in the presence of 150 mg/mL ficoll, droplet formation was suppressed by DNAs, indicating a strong inhibitory effect of DNAs in droplet formation (Fig. 5h).

In order to examine the effect of p53 posttranslational modifications on protein activation, we investigated droplet formation on a S392E mutant that mimicked phosphorylation of the 392^nd^ residue. The S392E mutation suppressed droplet formation in 45 mM NaCl at pH 7.0, suggesting an enhanced electrostatic repulsion by the phosphor-mimic mutation (Fig. 5i). Accordingly, this posttranslational modification might be a key factor that regulates p53 droplet formation.

## Discussion

In this study, we found that p53 itself can form liquid-like droplets at slightly acidic and neutral pHs and at low salt concentrations. These droplets are highly fluidic, as exemplified by fusion of multiple droplets and by the uniform distribution of p53 within them. We identified that the NT and CT disordered domains mainly participate in p53 droplet formation. FRET measurements demonstrated that the distances between core domains and between CT domains in the p53 dimer are altered due to intermolecular interactions in the droplet. Molecular crowding enhances p53 droplet formation, whereas DNA and ATP dissolve the droplets. The target binding of p53 dissolved from the spherical droplets at pH 7.0 can be fully restored. Finally, we found that the p53 S392E mutant, mimicking the 392^nd^ residue phosphorylation, does not form droplets. These observations demonstrate that p53 itself is an example of proteins that exhibit LLPS. In this section, we discuss the molecular mechanism of p53 droplet formation and the physiological implications of the current findings.

**Figure 6:**
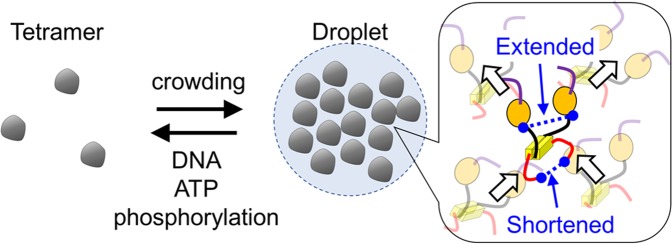
Schematic diagram of the phase separation phenomenon for p53. The LLPS of p53 is enhanced by the crowding effect. In contrast, DNA, ATP, and phosphorylation dissolve p53 droplets. p53 is composed of the NT (purple), Core (orange), Tet (yellow) and CT (red) domains. In the droplets, the NT and CT domains interact electrostatically. Arrows in the inset denote the structural changes on the different domains of p53 that are induced by the intermolecular interactions within the droplet. The dimer structure is displayed for clarity.

We propose that p53 promotes droplet formation based on multivalent electrostatic interactions among the NT, core, and CT domains (Fig. 6). This proposal is based on the observations that droplet formation was suppressed at higher salt concentrations (Fig. 1b,c) and that the deletion of the NT domain, of the NT and core domains, or of the CT domain suppressed droplet formation (Fig. 2). The net charges per residue in the NT and CT domains are at least 1.7-fold larger than those in the other domains (Supplementary Table S1). Considering the large and opposite charges of the NT and CT domains, electrostatic interaction should dominantly stabilize p53 droplet formation. The effect of DNA on droplet formation can similarly be explained by the electrostatic effect, since DNA bound to p53 should negatively shift the net charge of the complex, increasing electrostatic repulsion among p53-DNA complexes (Fig. 5). Within the droplets, the core domains might be pulled by the interacting CT domains of other p53 molecules, extending the distance between core domains (Figs. 3f and 6). In contrast, the distance between disordered CT domains is shortened likely due to the interaction with the NT and/or core domains (Figs. 3g and 6). In cells, p53 forms dimeric and/or tetrameric conformations possessing at least two sets of NT, core and CT domains^56^, which enable multivalent electrostatic interactions among p53 proteins and leading to the formation of micrometer-sized droplets.

The LLPS induced by the multiple oppositely-charged domains of p53 is unique for this protein and is distinct from the well-known LLPS phenomenon promoted by the low complexity sequence region of FUS^57^, Laf-1^58^, RNA polymerase^50^, TDP43^59^, EWSR1^59^, TAF15^59^, and hnRnPA1^59^. In the latter proteins, the low complexity sequence region provides cation-π interaction^60,61^ and/or π-π interaction^62^, stabilizing the droplets. The LLPS predictor, which is based on machine learning of the π-π interaction in amino acid sequences, gave a moderate score for p53. This is lower than the scores for the above proteins showing LLPS, supporting the distinct stabilization mechanism of the p53 droplets^62^. In general, electrostatic interactions are known to be the key interactions for LLPS formation upon the mixing of two components^63–67^. Thus, p53 might be the first protein that induces LLPS using two oppositely-charged domains in a single component system.

The current results demonstrate that p53 can form liquid-like droplets *in vitro* and suggest the possibility that these droplets might also be formed in physiological cellular conditions. Cytosol pH and salt concentration are ~7.4 and ~150 mM, respectively. The cellular p53 concentration is approximately 18–180 nM, calculated using the protein’s copy number per cell and the nucleic volume considering a 10 μm diameter^68^. In the condition mimicking the cellular environment by adding 150 mg/mL ficoll and 145 mM NaCl at pH 7.0, we detected increase in the scattering intensity at the p53 concentration as low as 1 μM, showing the formation of submicrometer-sized droplets (Fig. S5a). Furthermore, the fluorescence microscopy observation of the same solution containing 0.18 μM p53 labeled with alexa488 revealed highly fluorescent spots, further demonstrating the formation of submicrometer-sized assembles at a possible cellular concentration of p53 (Fig. S5b). In earlier studies, the *in vivo* fluorescence imaging of p53 conjugated with a fluorescent protein shows heterogeneous distribution of p53 as well as spherical clusters in the cell^69–73^. Accordingly, the formation of micron-sized or submicrometer-sized assemblies of p53 in cells is a likely possibility at a physiological condition.

We suggest that the liquid droplets of p53 might work as a functional switch (Fig. 6). When p53 forms the droplets, the target search and binding might be retarded due to the lowing of the dispersed tetramer concentration. If p53 is activated by the posttranslational modification and/or the binding by cellular components under some stresses to the cell, p53 might be released from the droplets, search for, and bind to the target DNA sequence, regulating the transcriptional expression of downstream genes. The suggestion is supported by the results that p53 can bind to the target DNA after dissolution of the droplets and that p53 phosphorylation suppresses the droplet formation (Figs. 4 and 5). Accordingly, we propose that the compartmentalization of p53 into the droplets in the normal cell condition suppresses the function of p53 as transcriptional regulator, and that the activation of p53 under the stress conditions releases p53 from the droplets and promotes the function. Further investigation is necessary to clarify the roles of p53 in cellular conditions including the possible involvement in the other cellular assembles such as the Cajal and PLM bodies.

## Materials and Methods

### p53 mutants

We prepared FL-p53 as well as the TetCT, NTCoreTet, and S392E mutants as described previously^5,8^. For FL-p53, the thermostable and cysteine-modified mutant of human p53 (C124A, C135V, C141V, W146Y, C182S, V203A, R209P, C229Y, H233Y, Y234F, N235K, Y236F, T253V, N268D, C275A, C277A, K292C) was used^5^. The TetCT mutant corresponds to residues 293−393 of FL-p53 with an additional N-terminal cysteine^5^. The NTCoreTet mutant corresponds to residues 1–363^6^. The S392E mutant was constructed based on FL-p53^8^. We prepared the CoreTetCT mutant, corresponding to residues 94–393. The CoreTetCT gene was cloned in plasmid pGEX-6P-1 using an In-Fusion HD Cloning Kit (Clontech, Mountain View, CA). The two dimer FL-p53 mutants, L344A and L344A/C292K/394 C, were newly prepared for FRET measurements. The dimer mutant genes in pGEX-6P-1 were generated using a PrimeSTAR Mutagenesis Basal Kit (TaKaRa, Shiga, Japan). Expression and purification of the six mutants were conducted as previously described^5,6^. We confirmed the dimerization of the L334A mutant and the tetramerization of FL-p53 using a gel filtration column (Superdex 200; GE Healthcare, Tokyo, Japan).

### Labeling of p53 mutants with fluorophores

FL-p53 was labeled with Alexa488 (Thermo Fisher, Tokyo, Japan) using maleimide chemistry and was further purified with a cation exchange column^5^. The p53 dimer mutants were labeled with Alexa488 or Alexa594 (Thermo Fisher) and purified by a cation exchange column for FRET measurements^5^. The labeling ratios were determined by the absorbances at 280 nm and at 495 or 590 nm.

### Sample solutions for the droplet formation experiments

The scattering and DIC measurements used solutions containing 12 μM FL-p53, 20 mM Hepes or Mes, various concentrations of NaCl, 0.5 mM EDTA, and 1 mM DTT at pHs 5.5–8.0. These solutions were prepared through a 10-fold dilution of a stock solution containing 450 mM NaCl at pH 7.5. The investigations on additives such as DNAs and ATP used a solution containing 10 μM FL-p53, 20 mM Hepes, 45 mM NaCl, 0.5 mM EDTA, and 1 mM DTT at pH 7.0. The specific DNA sequence was 5′-ATCAGGAACATGTCCCAACATGTTGAGCTC-3′, which corresponds to the p21 5′ promoter site^8^. The non-specific DNA sequence was 5′-AATATGGTTTGAATAAAGAGTAAAGATTTG-3′^8^. The ssDNA sequence was 5′-ATCAGGAACATGTCCCAACATGTTGAGCTC-3′. The DNA fragments and λDNA were purchased from SigmaAldrich and New England Biolabs, respectively. For the investigation of the molecular crowding effect, we used a solution containing 10 μM FL-p53, 20 mM Hepes, 145 mM NaCl, 0.5 mM EDTA, 1 mM DTT, and 0–150 mg/mL ficoll or dextran at pH 7.0. We used ficoll PM400 with an average molecular weight of 400,000 (GE Healthcare) or Dextran with a molecular weight between 45,000 and 65,000 (Sigma-Aldrich, Tokyo, Japan). For the investigations of the S392E mutant, we used a solution containing 12 μM of the S392E mutant or of FL-p53, 20 mM Hepes, 45 mM NaCl, 0.5 mM EDTA, and 1 mM DTT at pH 7.0.

The fluorescence and DIC microscopies used a solution containing 0.12 μM Alexa488-labeled FL-p53, 12 μM FL-p53, 20 mM Hepes, 45 mM NaCl, 0.5 mM EDTA, 1 mM DTT, and 150 mg/mL Dextran at pH 7.0. For the incorporation of dimer mutants into the FL-p53 droplets, we prepared a solution containing 0.12 μM of the dimer mutant (L344A) labeled at 292 C by Alexa488 or Alexa594, 12 μM FL-p53, 20 mM Hepes, 45 mM NaCl, 0.5 mM EDTA, 1 mM DTT, and 150 mg/mL Dextran at pH 7.0.

When performing the microscope-based FRET measurements, we incubated 0.18 μM of the Alexa488-Alexa488 dimer labeled at 292 C with 0.82 μM of the Alexa594-Alexa594 dimer labeled at 292 C in a solution containing 20 mM Hepes, 45 mM NaCl, 0.5 mM EDTA, 1 mM DTT, and 150 mg/mL Dextran at pH 7.0 for 20 min. We also incubated 13 μM of the non-labeled FL-p53 in the same solution for 20 min. We then mixed the two solutions resulting in apparent concentrations of the Alexa488-Alexa488 dimer, the Alexa594-Alexa594 dimer, and of FL-p53 at 0.023 μM, 0.12 μM, and 12 μM, respectively. Corresponding experiments were similarly performed using the dimer mutant labeled at 394 C (L344A/C292K/394 C).

For the microscope-based FRET measurements of the dimers in solution, we incubated 0.3 μM of the Alexa488-Alexa488 dimer labeled at 292 C and 1.5 μM of the Alexa594-Alexa594 dimer labeled at 292 C in a solution containing 20 mM Hepes, 45 mM NaCl, 0.5 mM EDTA, 1 mM DTT, and 150 mg/mL Dextran at pH 7.0 for 20 min. Corresponding experiments were similarly performed using the dimer mutant labeled at 394 C (L344A/C292K/394 C). The concentrations of p53 mutants per monomer were used through the manuscript.

### Scattering measurements

We measured scattering of the droplets for p53 mutants as the OD_350_ at 23°C using an absorbance spectrometer (Nano Drop One; Thermo Fisher). The optical path length was 1 mm. The OD_350_ values were displayed for a path length of 10 mm.

### DIC microscopy

We used the DIC detection mode of the inverted microscope (IX-73; Olympus, Tokyo, Japan) equipped with a microscopic objective (LCPlanFL, NA = 0.6, 40 × ). A mercury lamp (U-HGLGPS; Olympus) and a color-sensitive camera (DP73; Olympus) were used as the light source and the detector, respectively. We used a flow cell composed of the coverslip (Matsunami Glass, Osaka, Japan), a double-sided tape with 100 μm thickness, and the slide glass (Matsunami Glass). The coverslip and slide glass were cleaned with ethanol and 5 M KOH before use. In the fusion experiments, we coated the coverslip with 0.5% MPC polymer (Lipidureμ-CM5206; NOF Corp., Tokyo, Japan) in ethanol. The p53 solution was introduced into the flow cell and the DIC images were taken at 21°C. The DIC images were analyzed using ImageJ software. The color images were converted into merged images. The images in the absence of p53 were subtracted from those depicting p53 droplets. We then selected droplets with an area bigger than 0.2 μm^2^. The area and circularity were calculated for the selected droplets.

### Fluorescence anisotropy measurements

The fluorescence anisotropy of specific DNA strands labeled with 6-FAM was measured at 25°C using a fluorescence spectrometer (FP-6500, JASCO Co., Tokyo, Japan) with an automatic titrator and home-build autorotating polarizer^5^. We incubated solutions containing 12 μM FL-p53, 20 mM Hepes or Mes, 45 mM NaCl, 2 mM MgCl_2_, 0.5 mM EDTA, and 1 mM DTT at pHs 5.5, 7.0, and 7.9 for 25~30 mins. Then, we diluted the p53 solution for 5-fold into the solution containing 20 mM Hepes, 100 mM NaCl, 2 mM MgCl_2_, 0.5 mM EDTA, 1 mM DTT, and 0.1 mg/mL BSA to adjust pH to 7.9. FL-p53 was titrated into a solution containing 5 nM 6-FAM-labelled dsDNA_sp_, 20 mM Hepes, 90 mM NaCl, 2 mM MgCl_2_, 0.5 mM EDTA, 1 mM DTT, and 0.1 mg/mL BSA at pH 7.9. The detail of the curve fitting procedure was described in our previous report^5^.

### Fluorescence spectroscopy

For the investigations on the monomer exchange reaction for the dimer mutants, we used a fluorescence spectrometer (F-2500; Hitachi High Technologies, Tokyo, Japan). The excitation wavelength was 480 nm. The solution contained 20 mM Hepes, 50 mM KCl, 2 mM MgCl_2_, 1 mM DTT, and 0.2 mg/mL BSA at pH 7.9. In order to prevent the adsorption of p53 to the cuvette, we coated it with the MPC polymer. After mixing 10 nM of the Alexa488-Alexa488 dimer and 80 nM of the Alexa594-Alexa594 dimer, both dissolved in the above solution, the time course of the fluorescence spectrum was measured at 25°C. We calculated FRET efficiency based on the spectrometric data, *E*_S_, using the equation:

1 $${E}_{S}=\frac{{I}_{a}}{\frac{{\varPhi }_{a}}{{\varPhi }_{d}}{I}_{d}+{I}_{a}},$$

where *I*_a_, *I*_d_, *Φ*_a_ and *Φ*_d_ denote the fluorescence intensities and the quantum yields of the acceptor and the donor, respectively. The wavelength dependence of the spectrometer detection efficiency was corrected as previously described^74^. The maximum intensity of the spectrum of a pure Alexa488 solution was adjusted to that of the donor fluorescence in the observed FRET spectrum. *I*_d_ was then calculated as the integrated intensity from 490 nm to 700 nm in the spectrum of the pure Alexa488 solution. *I*_a_ was calculated as the integrated intensity from 490 nm to 700 nm after subtraction of the pure Alexa488 spectrum from the observed FRET spectrum for the sample.

### Fluorescence microscopy

We used the fluorescence detection mode of an inverted microscope (IX-73; Olympus). The mercury lamp (U-HGLGPS; Olympus) and a color-sensitive camera (DP73; Olympus) were used as the light source and the detector, respectively. The excitation wavelength was 470–490 nm for Alexa488 and FRET, and 520–550 nm for Alexa594. The emission wavelength was 515–550 nm for Alexa488, over 515 nm for FRET, and over 580 nm for Alexa594. Fluorescence images were taken at 21°C. The donor and acceptor intensities were recorded as the color camera’s green and red channels, respectively. These channels correspond to intensities at 515–580 nm and at 580–640 nm, respectively. In order to reduce the background, we casted a p53 solution on the coverslip and covered it with a slide glass without using double-sided tapes.

The fluorescence images were analyzed using ImageJ software. The raw fluorescence images were background corrected by subtracting the images obtained for samples in the absence of the fluorescent-labeled p53, in the same observation condition. The estimation of FRET efficiencies for the dimer mutants within the droplets was performed selecting droplets that presented a cross-section area of at least 10.8 μm^2^ in the images and calculating intensities of the green and red channels, *I*_G_ and *I*_R_, averaged over the cross-section for each selected droplet. The apparent FRET efficiency of each droplet based on microscopic observation, *E*_M_, was calculated using the equation:

2 $${E}_{M}=\frac{{I}_{R}}{\frac{{\varPhi }_{a}}{{\varPhi }_{d}}{I}_{G}+{I}_{R}}.$$

*I*_G_ and *I*_R_ were used without correction for the wavelength dependence of the camera detection efficiency or for the leakage of donor intensity into the acceptor intensity. For the FRET in solution, we calculated the average intensities of the green and red channels in several randomly selected areas and obtained the *E*_M_ values of the labeled dimer mutants in solution.

## Supplementary information

Supplementary Information.
