# Supplementary material for: Liquid-like droplet formation by tumor suppressor p53 induced by multivalent electrostatic interactions between two disordered domains
Source: Sci Rep. 2020 Jan 17;10:580. doi: 10.1038/s41598-020-57521-w (PMC6969132; doi:10.1038/s41598-020-57521-w)
Supplement: Supplementary file 1 — Supplementary Information. [file 41598_2020_57521_MOESM1_ESM.pdf]

Supplementary Information for

Liquid-like droplet formation by tumor suppressor p53 induced by multivalent electrostatic interactions between two disordered domains

Kiyoto Kamagata[1,2,*], Saori Kanbayashi[1], Masaya Honda[1,2], Yuji Itoh[1], Hiroto Takahashi[1], Tomoshi Kameda[3], Fumi Nagatsugi[1,2], and Satoshi Takahashi[1,2]

[1]Institute of Multidisciplinary Research for Advanced Materials, Tohoku University, Katahira 2-1-1, Aoba-ku, Sendai 980-8577, Japan

[2]Department of Chemistry, Graduate School of Science, Tohoku University, Sendai 980-8578, Japan

[3]Artificial Intelligence Research Center, National Institute of Advanced Industrial Science and Technology (AIST), Koto, Tokyo 135-0064, Japan

*Corresponding author: Kiyoto Kamagata

Institute of Multidisciplinary Research for Advanced Materials, Tohoku University, Katahira 2-1-1, Aoba-ku, Sendai 980-8577, Japan

TEL: +81-22-217-5843/FAX: +81-22-217-5842

e-mail: kiyoto.kamagata.e8@tohoku.ac.jp

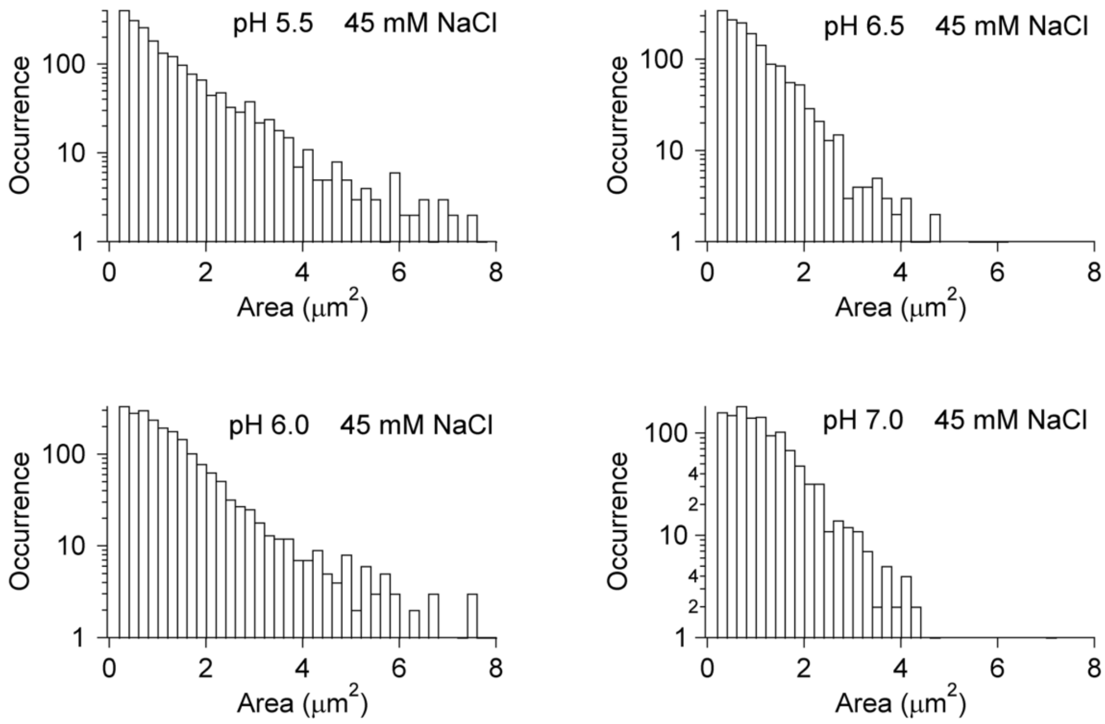

Fig. S1. Distributions of cross-section areas of individual FL-p53 droplets and/or clusters observed by DIC microscopy at various pHs. All solutions contain 10 μM FL-p53 and 45 mM NaCl.

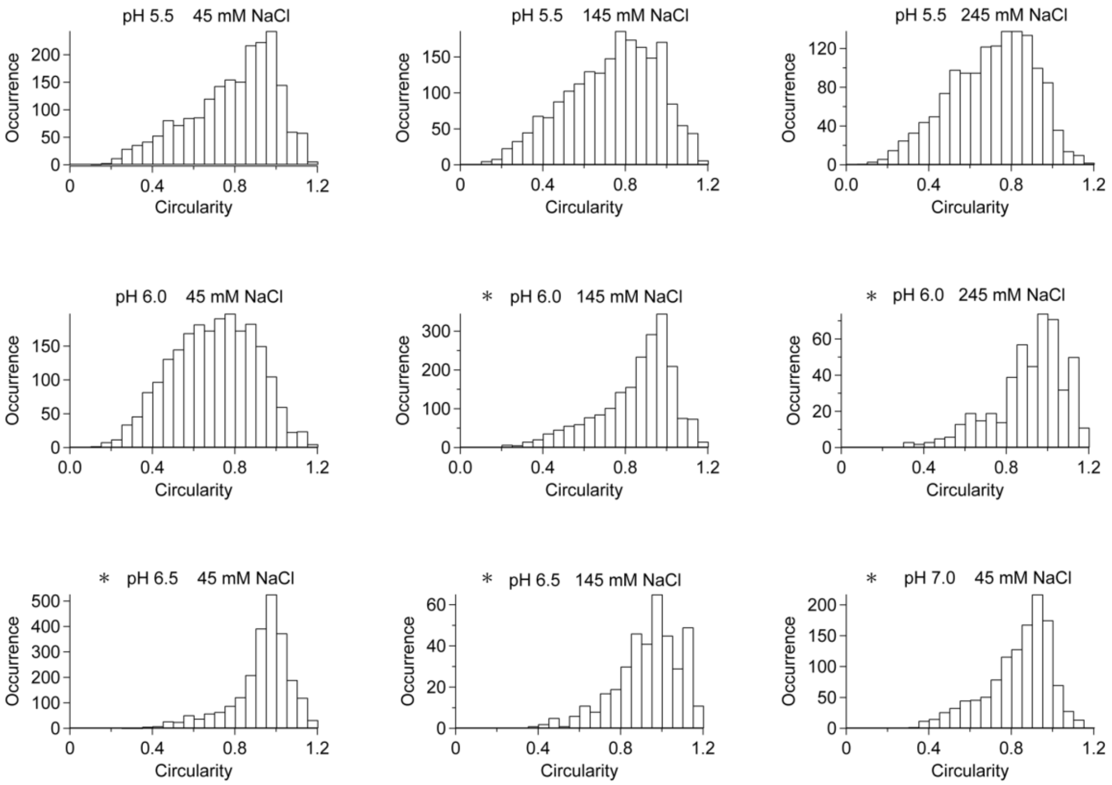

Fig. S2. Distribution of circularity of individual FL-p53 droplets and/or clusters at different pHs and salt concentrations. FL-p53 concentration was 12 μM. Asterisks (*) represent the conditions in which the main peak of circularity appeared around 1.

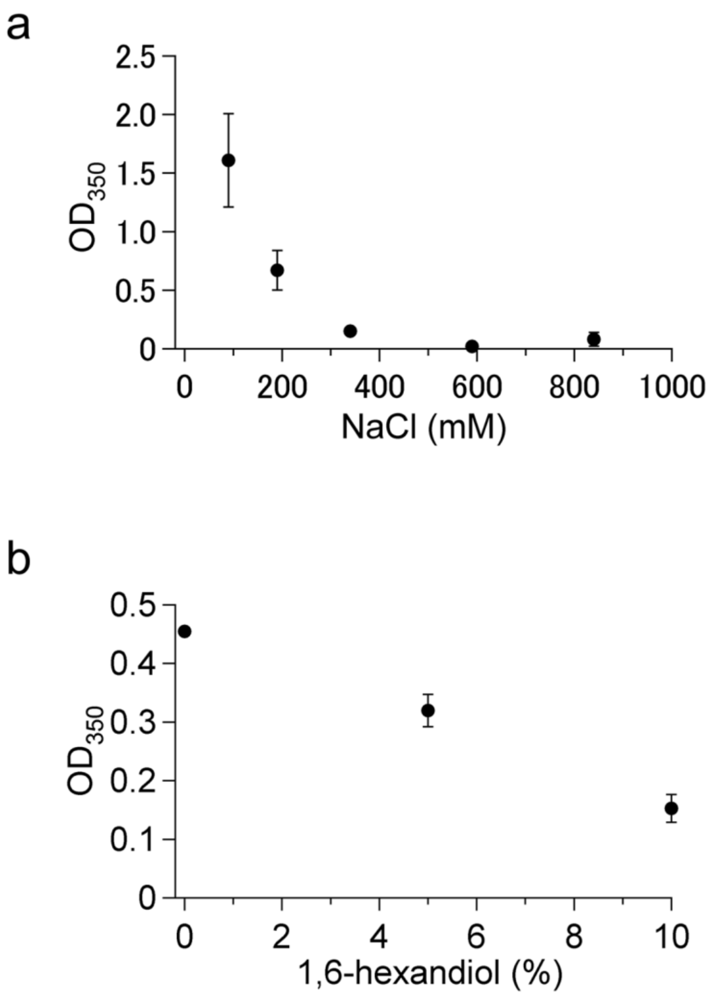

Fig. S3. (a) Salt-concentration dependence of droplet formation of 10 μM FL-p53 in 150 mg/mL Ficoll at pH 7.0. (b) Effect of 1,6-hexandiol on droplet formation of 10 μM FL-p53 in 45 mM NaCl at pH 7.0.

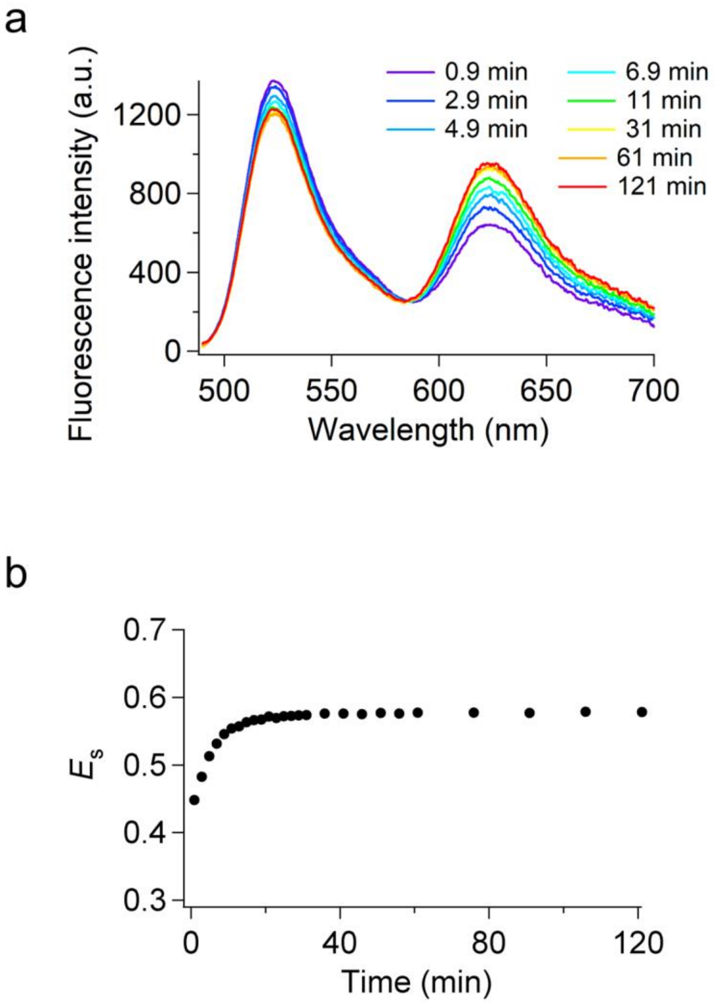

Fig. S4. (a) Fluorescence spectral changes observed after mixing the Alexa488-Alexa488 and the Alexa594-Alexa594 dimers, both labeled at 394C at pH 7.9 in the presence of 50 mM KCl. (b) Time course of the spectrum-based FRET efficiency, E_S, after mixing the Alexa488-Alexa488 dimer and the Alexa594-Alexa594 dimer labeled at 394C. The data shown in panel (a) were used for the calculation of E_S using Eq. 1.

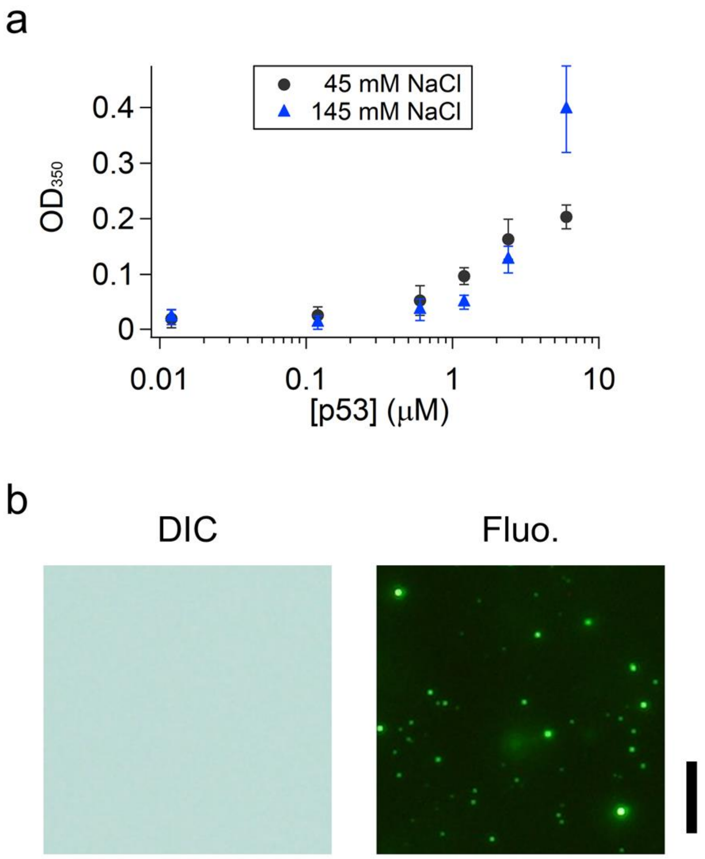

Fig. S5. (a) Dependence of the droplet formation on the concentration of FL-p53 in the condition containing 150 mg/mL Ficoll at pH 7.0. The scattering intensities at 350 nm in the presence of 45 mM or 145 mM NaCl were presented. (b) DIC and fluorescence microscopic images of the p53 droplets formed by 0.18 μM Alexa488-labeled p53 at pH 7.0 in the presence of 145 mM NaCl and 150 mg/mL dextran. Scale bar represents 10 μm. While micrometer-sized droplets were not observed in the DIC microscopic image, many spots with significant fluorescence intensities were observed in the fluorescence microscopic image, which may correspond to the submicrometer-sized droplets.

Table S1. Net charges in different p53 domains at pH 7.0.

domain	# of residues	Net charge$	Net charge per residue$
N-terminal	94	−13.7	−0.15
Core	198	4.8	0.02
Linker	33	2.5	0.08
Tetramerization	31	−2.0	−0.06
C-terminal	37	4.7	0.13

$The charge of amino acids was estimated using a simulated structure and PROPKA3.

Table S2. Labeling ratio of fluorophores to the p53 mutants.

p53 mutants	fluorophore	labeling ratio[#]
FL-p53	alexa488	0.78
p53 dimer (292C)	alexa488	1.04 or 1.05
p53 dimer (292C)	alexa594	0.99 or 1.33
p53 dimer (394C)	alexa488	0.80
p53 dimer (394C)	alexa594	1.00

[#]Labeling ratio per p53 monomer.

Supplementary text

Partial charge estimation of p53 at various pHs.

To understand pH dependency of LLPS in electrostatic viewpoint, we attributed to the partial charge of each domain of FL-p53. The pKa value of histidine ranges between 5 and 7, which mainly affect the partial charge of domains of p53. We estimated the pKa and partial charge value of amino acids of p53 using PROPKA3 [1]. The prediction by PROPKA requires the three dimensional structure. Firstly, we made the structure of FL-p53, NTCoreTet, CoreTetCT, and TetCT mutants by homology modelling based on SWISSMODEL [2]. Secondly, 100 ns molecular dynamics simulation was carried out, and the last structure was used as a query structure for PROPKA3. The p53 molecules were simulated using the AMBER ff14SB force field [3]. The solvation and ionic effect were modeled by the generalized Born energy [4]. The temperature was fixed at 298K, controlled using a Langevin thermostat with 1.0 ps^{-1} of the water viscosity. The noncovalent interactions, such as electrostatics, van der Waals, and generalized Born, were used without cutoffs. The simulations were conducted using the Amber18 simulator [5] with GPU [6].

Estimation of the distance between dyes and the FRET efficiency

To confirm if the FRET efficiency reflects the distance information, we estimated the mean distance between the dyes and compared the experimental and theoretical FRET efficiencies.

We first estimated the distance between the 292nd residues of the p53 dimer assuming a heterogeneous Gaussian chain with the folded dimerization domain and two disordered regions composed of residues 292-326. The distance between C_α in the 326th residues in the folded dimerization domain, d_{fold}, was calculated as 1.31 nm using the crystal structure (PDB ID: 1A1U). The mean distance between the 292nd residues, R, was calculated as 4.78 nm using the following equation:

$$R = \sqrt{0.55^2 \times 2 \times N + d_{fold}^2} \quad (nm),$$

where N is the number of residues in the disordered region. This equation was obtained with some modifications of the end to end distance for Gaussian chain in ref. [7]. We next calculated the mean FRET efficiency, $<E>$, using three following equations:

$$P(r) = \left(\frac{3}{2\pi}\right)^{3/2} \frac{4\pi}{R} \left(\frac{r}{R}\right)^2 \exp\left(-\frac{3}{2}\left(\frac{r}{R}\right)^2\right),$$

$$E(r) = \frac{1}{1 + (r/6)^6},$$

$$< \mathrm{E} > = \int_0^\infty P(r)E(r)dr,$$

where r, $P(r)$, and $E(r)$ are the distance between 292nd residues, the distance distribution, and the FRET efficiency, respectively [7]. The Förster distance was 6.0 nm for Alexa488 and Alexa594 pairs. $\langle E \rangle$ value for the 292nd residues of the p53 dimer was calculated as 0.76, which was consistent with 0.63 of the experimentally obtained E value.

Similarly, the mean distance between the 394th residues of the p53 dimer was calculated as 5.09 nm. d_{fold} for residues 354-394 was 2.43 nm. The $\langle E \rangle$ value for the 394th residues of the p53 dimer was 0.72, which was consistent with 0.58 of the experimentally obtained E value.

Overall, we conclude that the FRET efficiency reflected structure information for the p53 dimer.

References

1. Olsson, M. H., Sondergaard, C. R., Rostkowski, M. & Jensen, J. H. PROPKA3: Consistent Treatment of Internal and Surface Residues in Empirical pKa Predictions. *J. Chem. Theory Comput.* **7**, 525-537 (2011).

2. Waterhouse, A., et al. SWISS-MODEL: homology modelling of protein structures and complexes. *Nucleic Acids Res.* **46**, W296-w303 (2018).

3. Maier, J. A., et al. ff14SB: Improving the Accuracy of Protein Side Chain and

Backbone Parameters from ff99SB. *J. Chem. Theory Comput.* **11**, 3696-3713 (2015).

4. Nguyen, H., Roe, D. R. & Simmerling, C. Improved Generalized Born Solvent Model Parameters for Protein Simulations. *J. Chem. Theory Comput.* **9**, 2020-2034 (2013).

5. Case, D. A., et al.: Amber 2018. University of California, San Francisco, (2018).

6. Gotz, A. W., et al. Routine Microsecond Molecular Dynamics Simulations with AMBER on GPUs. 1. Generalized Born. *J. Chem. Theory Comput.* **8**, 1542-1555 (2012).

7. Zheng, W., et al. Inferring properties of disordered chains from FRET transfer efficiencies. *J. Chem. Phys.* **148**, 123329 (2018).
